# Supplementary figures and images for: Chromatin activity of IκBα mediates the exit from naïve pluripotency
Source: eLife. 2025 Oct 22;14:RP102784. doi: 10.7554/eLife.102784 (PMC12543326; doi:10.7554/eLife.102784)

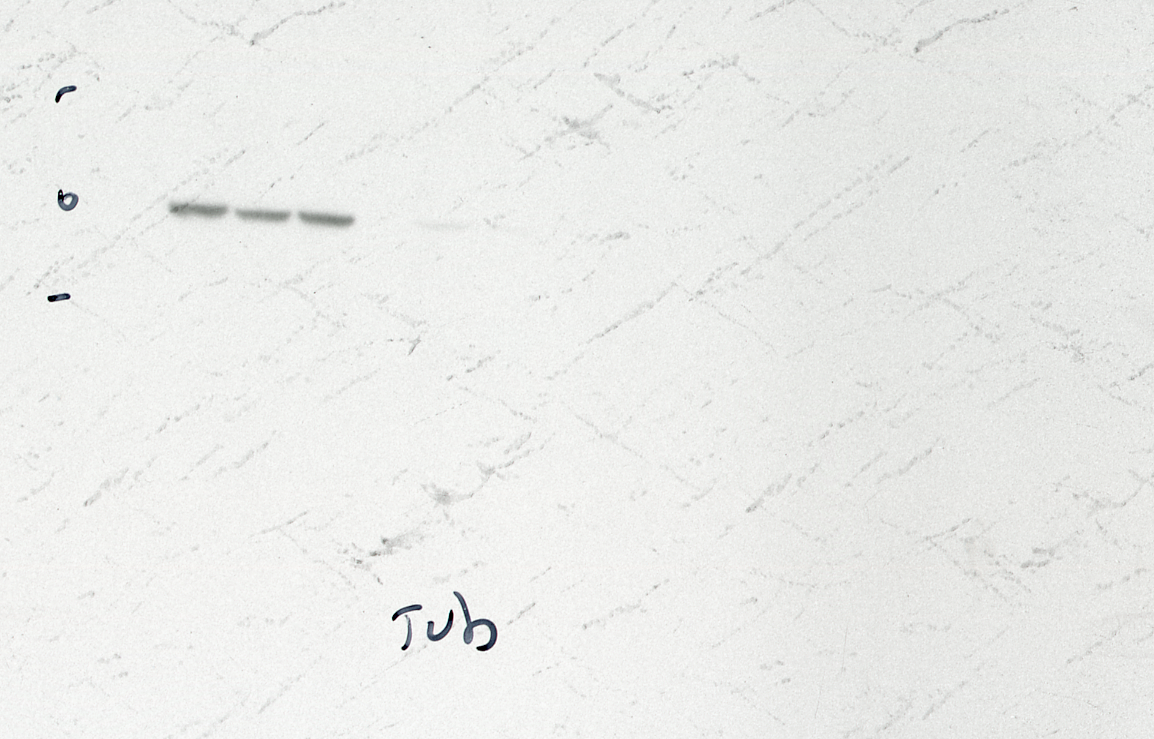

Supplement: Figure 1—source data 2. [file elife-102784-fig1-data2.zip › Figure 1_source data 2/Figure1D-raw-tub_2i_sl_epi020.tif]

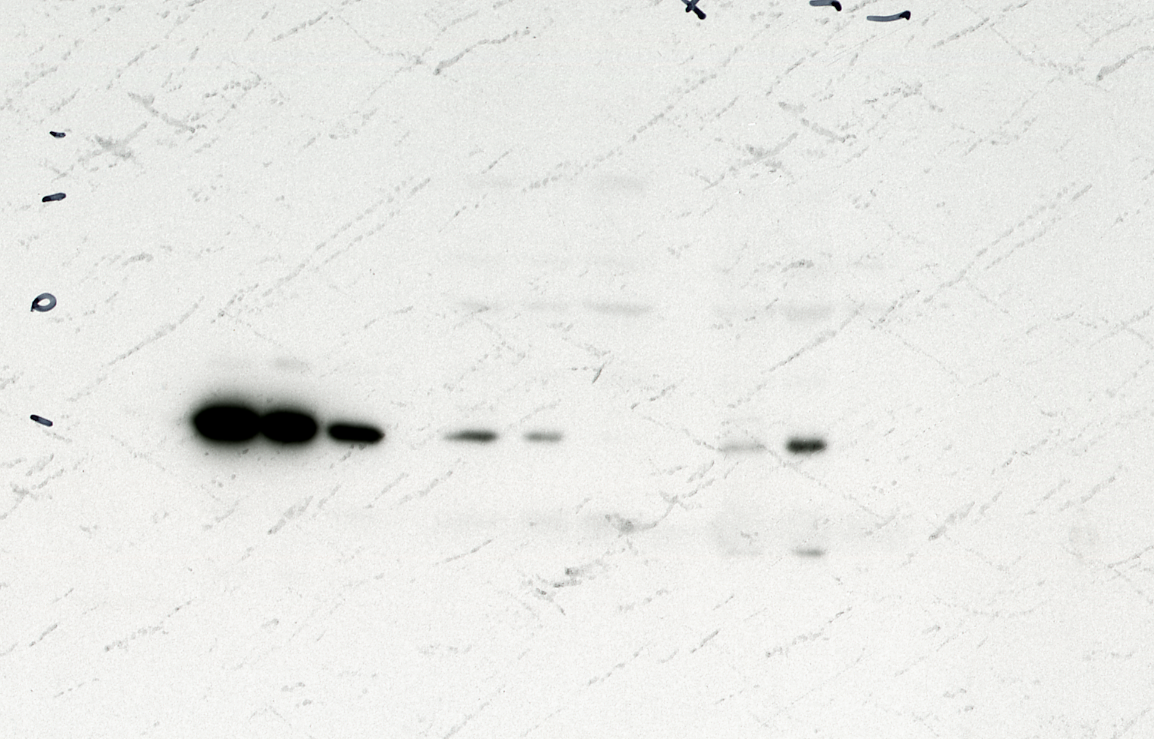

Supplement: Figure 1—source data 2. [file elife-102784-fig1-data2.zip › Figure 1_source data 2/Figure1D-raw-high_ikba-flag_2i_sl_epi022.tif]

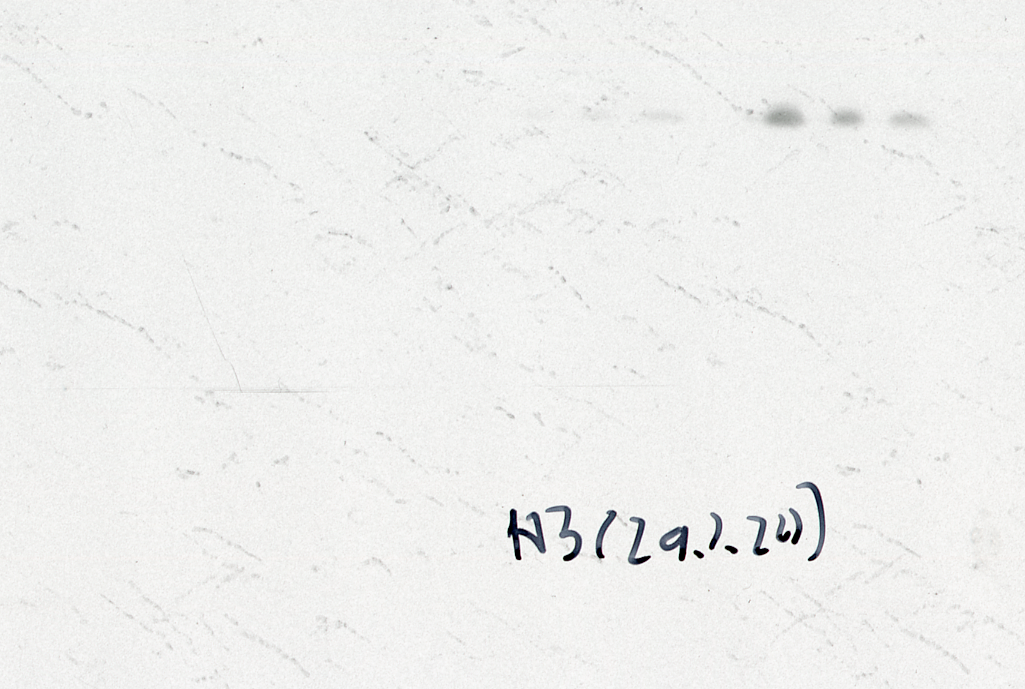

Supplement: Figure 1—source data 2. [file elife-102784-fig1-data2.zip › Figure 1_source data 2/Figure1D-raw-h3_2i_sl_epi018.tif]

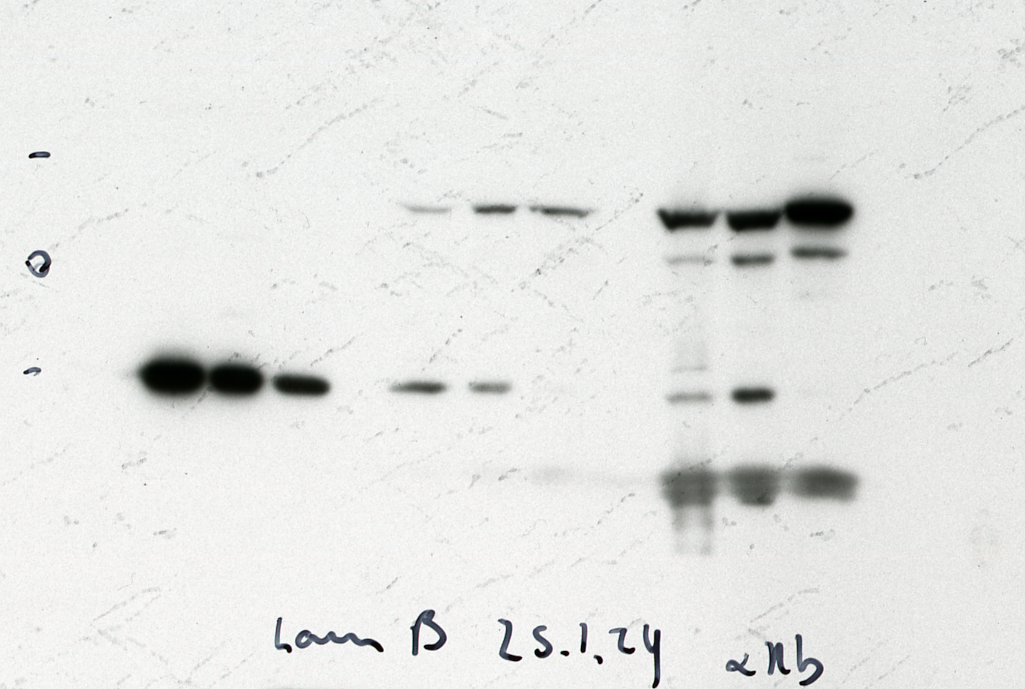

Supplement: Figure 1—source data 2. [file elife-102784-fig1-data2.zip › Figure 1_source data 2/Figure1D-raw-laminb_2i_sl_epi017.tif]

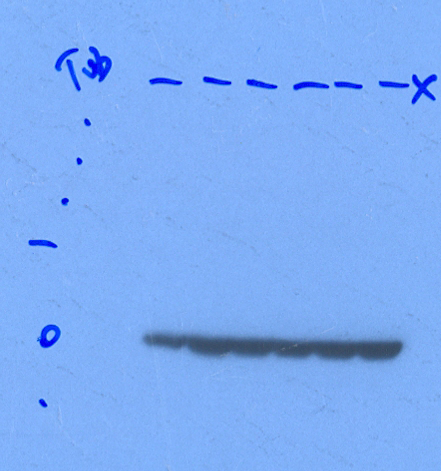

Supplement: Figure 2—figure supplement 1—source data 2. [file elife-102784-fig2-figsupp1-data2.zip › Figure2 figure supplement1_source data 2/Figure2S1-raw-tubulin_wt_ko_mESCs.tif]

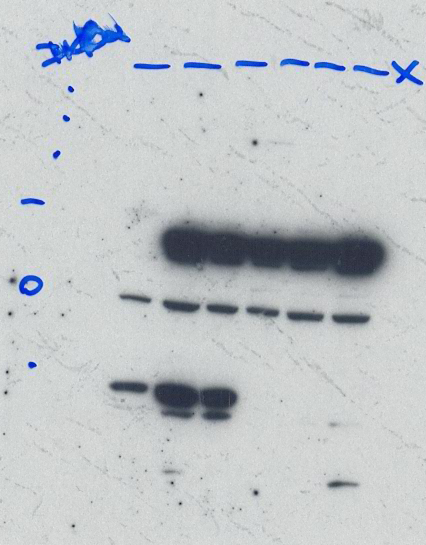

Supplement: Figure 2—figure supplement 1—source data 2. [file elife-102784-fig2-figsupp1-data2.zip › Figure2 figure supplement1_source data 2/Fgure2S1-raw-total_ikba_wt_kos_mESCs.tif]

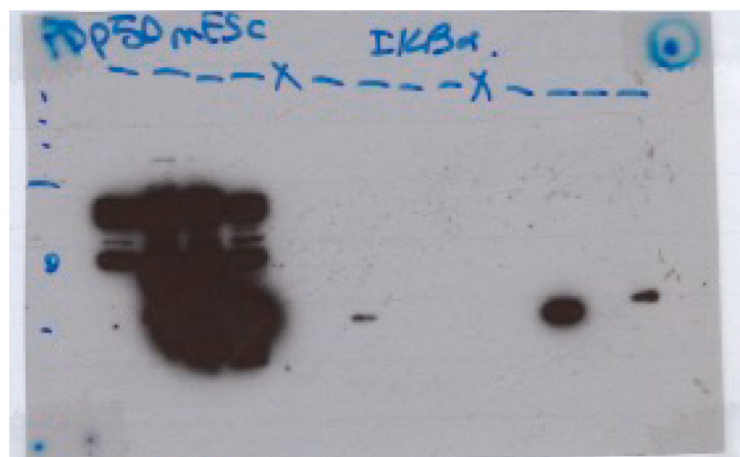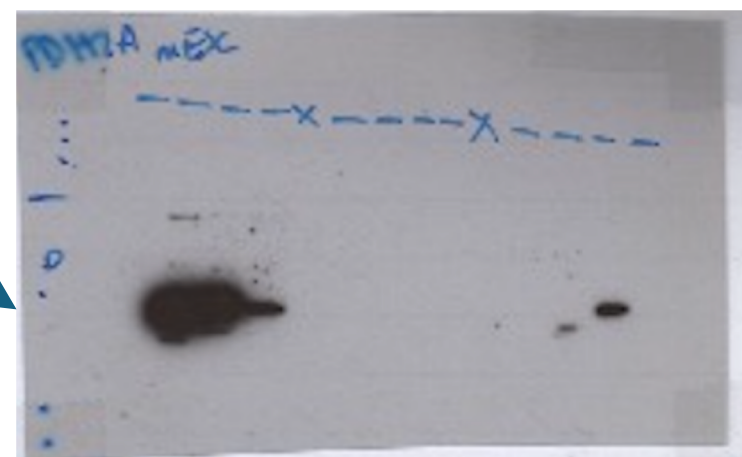

IκBα

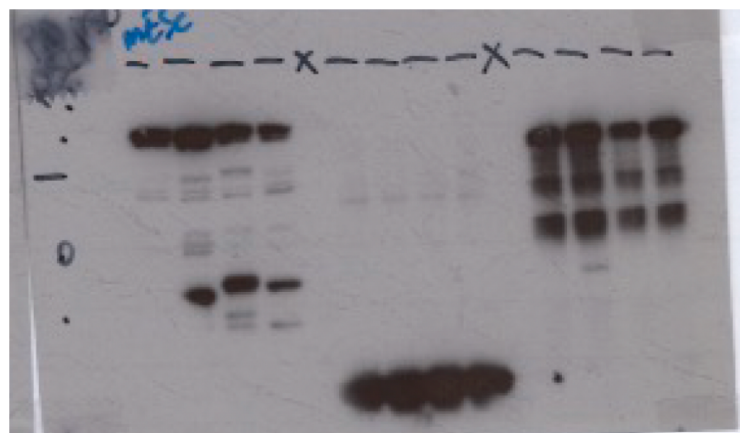

GSP-p50'

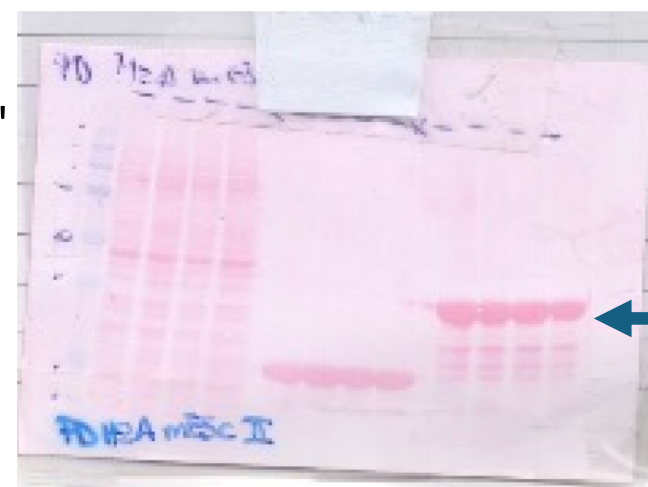

GST-H2A

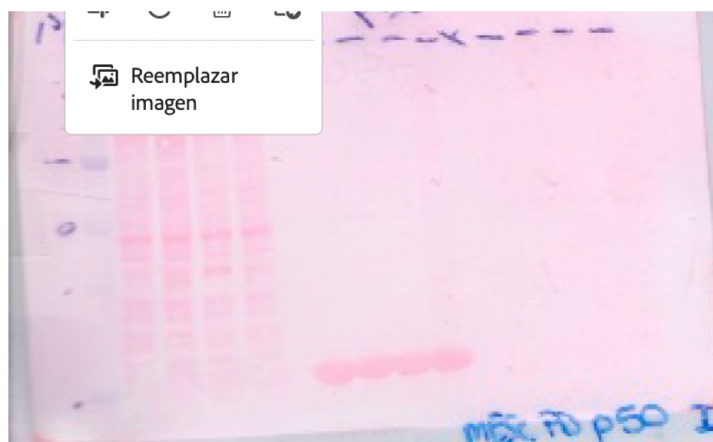

Figure 5. Source data 1. Original membranes corresponding to Figure 5, panel C.

Supplement: Figure 5—source data 1. [file elife-102784-fig5-data1.zip › Figure 5_source data 1/Figure5C-annotated.pdf]

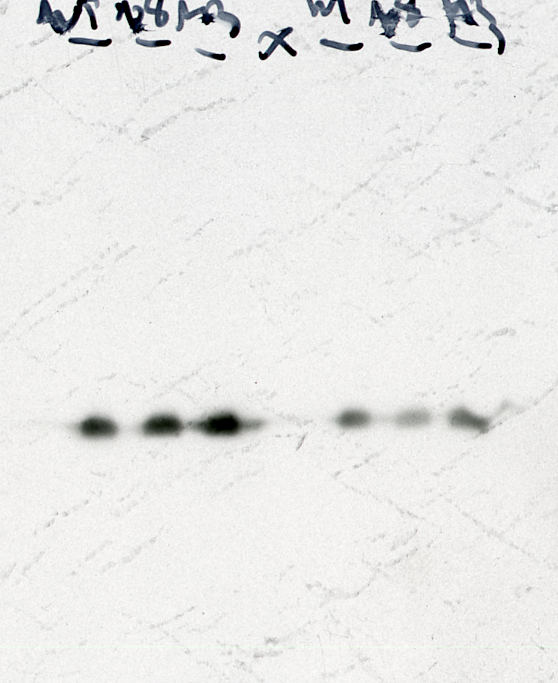

Supplement: Figure 5—source data 2. [file elife-102784-fig5-data2.zip › Figure 5_ source data 2/Figure 5E-raw data/Figure5E-raw-h3_iWT_i108_iM3.tif]

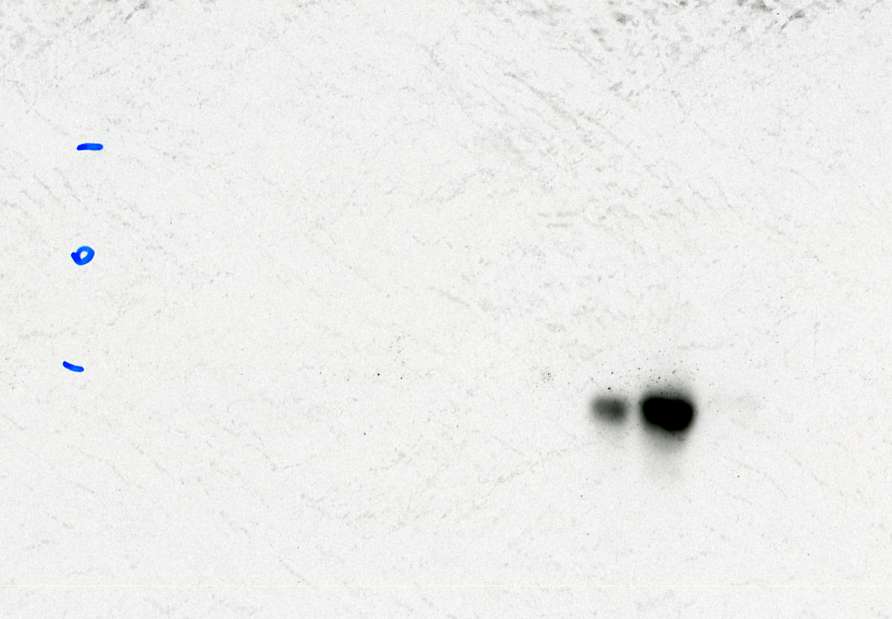

Supplement: Figure 5—source data 2. [file elife-102784-fig5-data2.zip › Figure 5_ source data 2/Figure 5E-raw data/Figure5E-raw higher_IkBa_iWT_i108_iM3.tif]

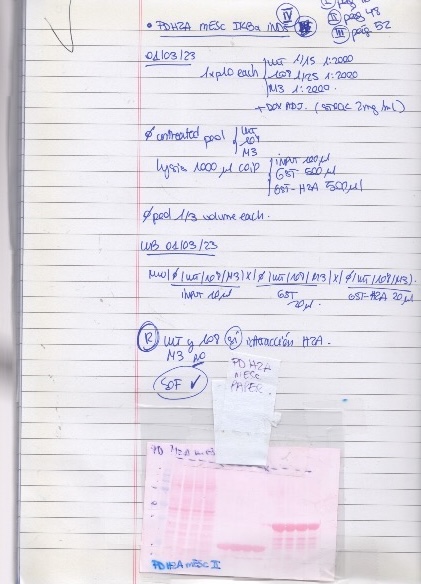

Supplement: Figure 5—source data 2. [file elife-102784-fig5-data2.zip › Figure 5_ source data 2/figure5C_raw data/Figure5C-raw loading-punceau.jpeg]

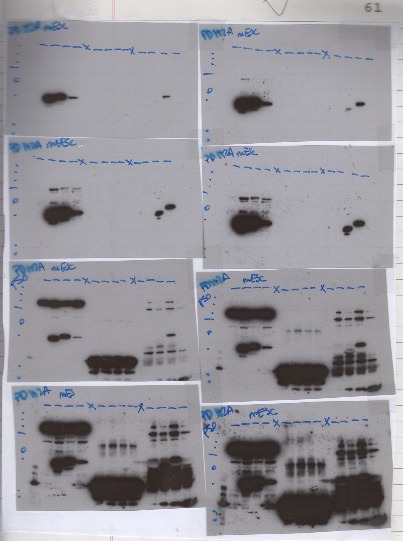

Supplement: Figure 5—source data 2. [file elife-102784-fig5-data2.zip › Figure 5_ source data 2/figure5C_raw data/Figure5C-rawH2A.jpeg]

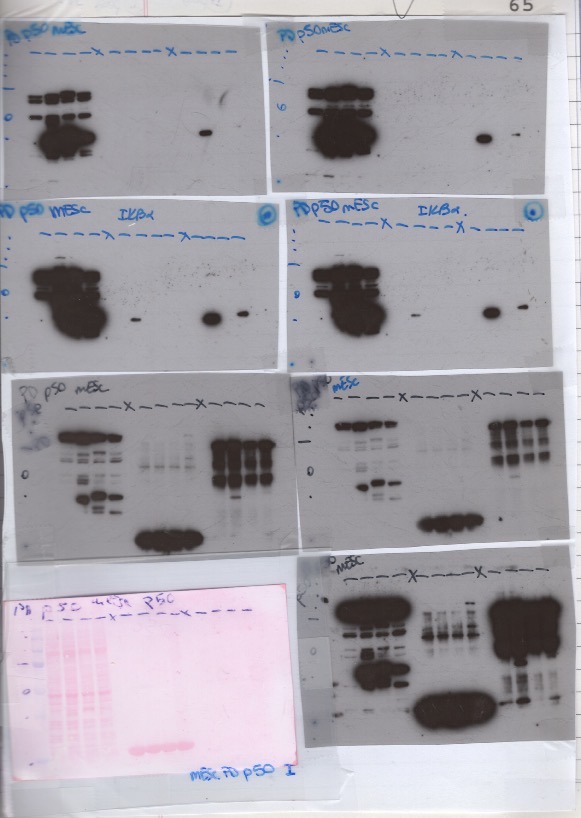

Supplement: Figure 5—source data 2. [file elife-102784-fig5-data2.zip › Figure 5_ source data 2/figure5C_raw data/Figure5C-rawikba.jpeg]

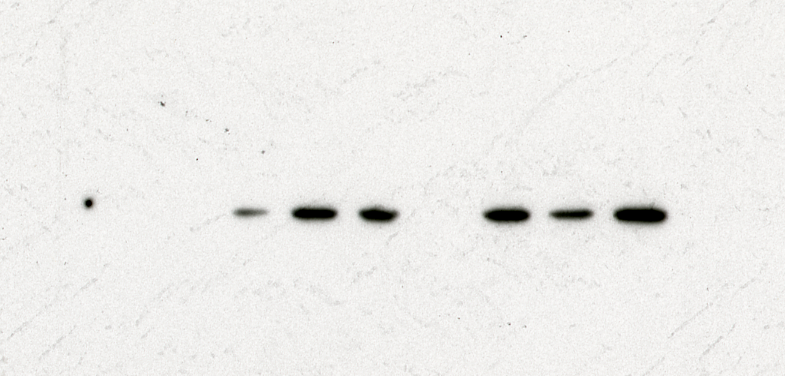

Supplement: Figure 5—source data 2. [file elife-102784-fig5-data2.zip › Figure 5_ source data 2/Figure5D-raw data/Figure 5D-raw-tubulin_iWT.tif]

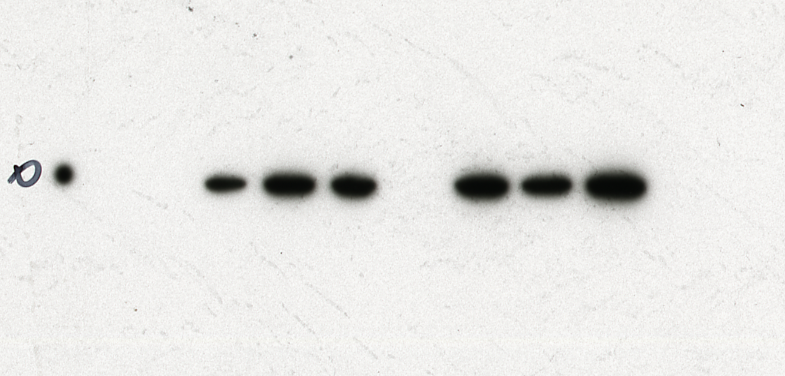

Supplement: Figure 5—source data 2. [file elife-102784-fig5-data2.zip › Figure 5_ source data 2/Figure5D-raw data/Figure 5Draw-high_tubulin.tif]
