# Supplementary material for: Chromatin activity of IκBα mediates the exit from naïve pluripotency
Source: eLife. 2025 Oct 22;14:RP102784. doi: 10.7554/eLife.102784 (PMC12543326; doi:10.7554/eLife.102784)
Supplement: Figure 5—source data 1. [file Figure 5D_annotated.pdf › Figure 5_source data 1/Figure 5D_annotated.pdf]

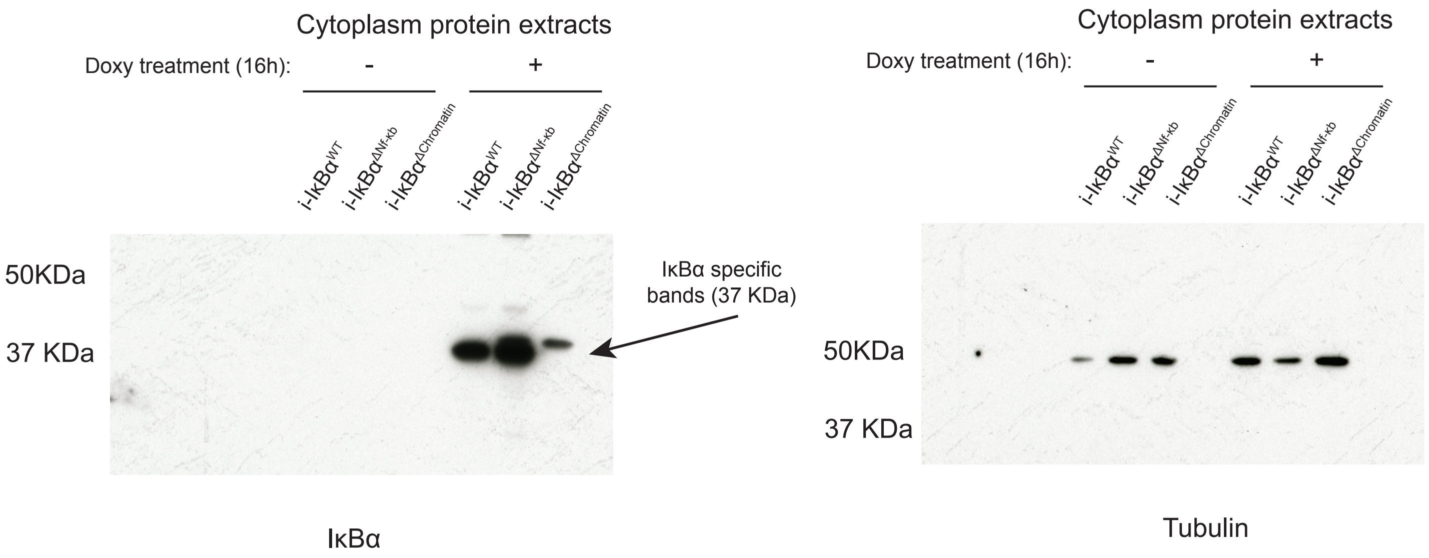

Figure 5. Source data 2. Original membranes corresponding to Figure 5, panel D. Only cytoplasmic protein extracts were loaded into the gel.
